# Neutrophil extracellular traps and their implications in airway inflammatory diseases

**DOI:** 10.3389/fmed.2023.1331000

**Published:** 2024-01-12

**Authors:** Nanxia Xuan, Jie Zhao, Zhiying Kang, Wei Cui, Bao-ping Tian

**Affiliations:** ^1^Department of Critical Care Medicine, The Second Affiliated Hospital, Zhejiang University School of Medicine, Hangzhou, China; ^2^Department of Critical Care Medicine, The First Affiliated Hospital of Ningbo University, Ningbo, China

**Keywords:** neutrophil extracellular traps, airway inflammation, ARDS, asthma, COPD, cystic fibrosis, targeted therapy

## Abstract

Neutrophil extracellular traps (NETs) are essential for immune defense and have been increasingly recognized for their role in infection and inflammation. In the context of airway inflammatory diseases, there is growing evidence suggesting the involvement and significance of NETs. This review aims to provide an overview of the formation mechanisms and components of NETs and their impact on various airway inflammatory diseases, including acute lung injury/ARDS, asthma, chronic obstructive pulmonary disease (COPD) and cystic fibrosis. By understanding the role of NETs in airway inflammation, we can gain valuable insights into the underlying pathogenesis of these diseases and identify potential targets for future therapeutic strategies that either target NETs formation or modulate their harmful effects. Further research is warranted to elucidate the complex interactions between NETs and airway inflammation and to develop targeted therapies that can effectively mitigate their detrimental effects while preserving their beneficial functions in host defense.

## 1 Introduction

Neutrophils, the most common type of white blood cells in mammalian blood, play a crucial role as the first line for defensing against infection and the non-specific immune response. They are activated and rapidly migrating to the site of infection through the blood vessel wall, guided by the cytokines and chemotactic factors. Upon activation, neutrophils not only phagocytose pathogens but also undergo a form of programmed cell death called NETosis, releasing a fibrous network known as neutrophil extracellular traps (NETs) (1, 2). NETs consist of genomic DNA as a scaffold, along with various proteins such as histones, myeloperoxidase (MPO), neutrophil elastase (NE), citrullinated histone 3 (CitH3), and cathepsin G (CG) (3). The web-like structure of NETs helps localize bacteria, fungi, viruses, and other pathogens to a specific area, slowing down the further spread of inflammation, however, research has shown that neutrophil extracellular traps are involved in the pathophysiology of various diseases. These include microbial infections, autoimmune diseases, inflammation, tumors, atherosclerosis, intracerebral hemorrhage, and thrombosis (4–13). Besides, age may also be associated with the formation of NETs. It has been reported that neutrophils from elderly patients respond to the presence of mitochondria, enhancing the formation of extracellular traps. It has also been found that these NETs formed by neutrophils in the elderly exhibit higher levels of oxidation and increased resistance to degradation by DNase I. Additionally, higher concentrations of residual NETs have been detected in the plasma of older individuals (14). However, the exact mechanisms through which age regulates NET formation are not fully understood, but the increased risk may be related to lower degradation of NETs in elderly patients.

It is important to note that NETs themselves can also damage respiratory epithelial cells and endothelial cells, and excessive production of NETs can worsen inflammatory lung injury (15–17). For example, neutrophils from patients with ARDS exhibit enhanced activity and an increased ability to release NETs, leading to severe pathological changes such as alveolar wall damage, pulmonary interstitial edema, and congestion. Due to the unique potential of NETs components to cause tissue injury and regulate immune responses, researchers have recognized that targeting the components of NETs or the related pathway could be a potential approach for treating various diseases including airway inflammatory diseases. By analyzing recent research on NETs, particularly in the context of respiratory diseases, we can gain insights into the progress and controversies surrounding NETs. This understanding will help us further comprehend the role of NETs in the pathophysiology of respiratory diseases, unravel disease mechanisms, and explore new therapeutic targets and strategies.

## 2 Mechanisms of NETs formation

### 2.1 Suicidal/lytic NETosis

It has been shown that various stimulating factors can initiate or trigger the process of neutrophil extracellular traps formation (18–20). These factors include mitogen phorbol 12-myristate 13-acetate (PMA), cholesterol crystal, calcium ionophore A23187, nigericin, immune complexes, and pathogens such as fungi, bacteria, viruses. Neutrophils are stimulated by PMA, resulting in the release of calcium ions from the endoplasmic reticulum into the cytoplasm. This stimulation activates nicotinamide adenine dinucleotide phosphate (NADPH) oxidase, which in turn leads to the activation of protein kinase C (PKC) and the downstream Ras/Raf/MEK/ERK signaling pathway. As a result, reactive oxygen species (ROS) are generated through NADPH oxidase. ROS can directly facilitate the translocation of peptidylarginine deiminase 4 (PAD4) into the nucleus, where it modifies histones through citrullination, ultimately leading to chromatin dispersion (21–23). Besides, upon activation and ROS production, NE translocates from granules to the nucleus and cleaves histones, promoting chromatin decondensation. Subsequently, MPO binds to chromatin, further promoting chromatin decondensation. The synergistic action of NE and MPO enhances chromatin decondensation, leading to cell membrane rupture and release of NETs (24, 25). NE can also cleave gasdermin D (GSDMD) in the cytoplasm, activating it to form pores on the plasma membrane, nuclear membrane, and granule membrane. As a result, antimicrobial proteins attach to the decondensed chromatin, forming a mesh-like structure (26). Additionally, during NETosis, the cytoskeletal systems, including microtubules, microfilaments, and intermediate filaments, rapidly disassemble, leading to cell membrane rupture, cell lysis, and the release of NETs into the extracellular space (27).

**Figure 1:**
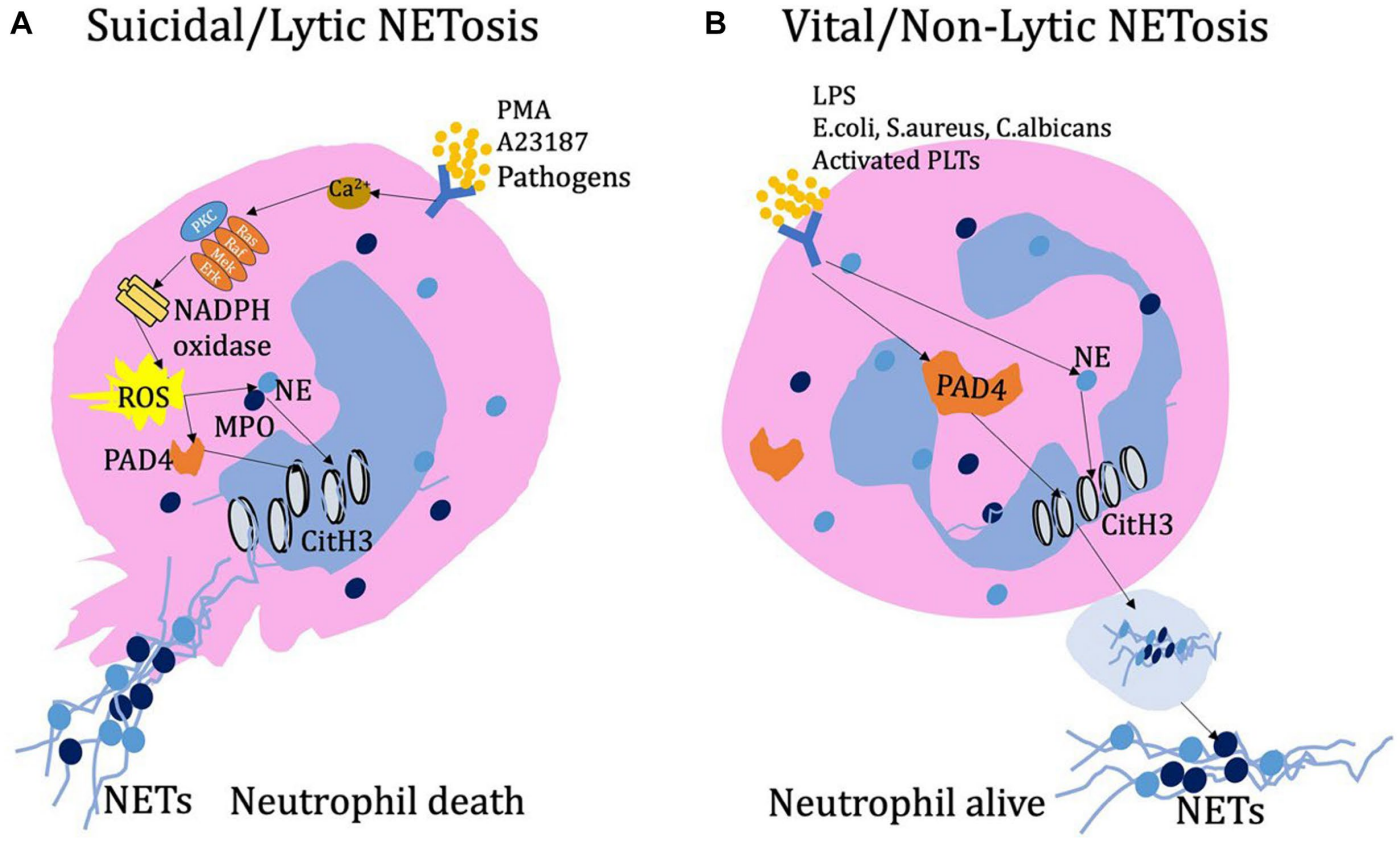
**(A)** Lytic NETs have been observed to form in response to various stimuli, including PMA, calcium ion carriers, and pathogens. When stimulated, neutrophils release Ca^2+^ ions and activate NADPH oxidase on the cell membrane. This activation can occur through the PKC or Ras-Raf-Mek-Erk signaling pathway, leading to the production of ROS. Subsequently, ROS promotes the entry of PAD4 into the nucleus, where it modifies histones through deamination, ultimately resulting in chromatin decondensation. Additionally, during activation and ROS production, the combined action of NE and MPO enhances chromatin decondensation. As NETosis progresses, the cell’s cytoskeleton system, including microtubules, microfilaments, and intermediate filaments, undergoes rapid disassembly. This disassembly ultimately leads to cell membrane rupture, cell dissolution, and the release of NETs into the extracellular space. **(B)** Early non-lytic NET formation occurs without causing cell lysis or death. This process is induced by various stimuli such as LPS, *E. coli*, *S. aureus*, *C. albicans*, or activated platelets. Simultaneously, the activation of PAD4 and the translocation of NE to the nucleus result in chromatin decondensation. The resulting protein-decorated chromatin is expelled via vesicles without disrupting the plasma membrane. Once released into the extracellular environment, NETs do not compromise the structural and functional integrity of neutrophils.

However, in reality, there are still different viewpoints regarding the NADPH oxidase-dependent NETs formation pathway. According to the research by Kenny et al. (28), the production of ROS is a hallmark of NETosis induced by PMA, *Candida albicans*, and Group B streptococcus. However, nigericin and A23187, in contrast, does not induce the production of ROS during the process of inducing NETs. Additionally, the ROS generated by NADPH oxidase is only partially required for the induction of NETosis by *C. albicans* and Group B streptococcus. During SARS-CoV-2 lung infection, the formation of NETosis is a key feature of severe COVID-19 (29, 30). Similarly, it is yet to be determined whether ROS directly participate in this process (31). Besides, the activation of neutrophil extracellular traps using the ionophores A23187 or nigericin does not depend on MPO or NE (28). Which provided the different opinions regarding the essential components of NETs during NETosis process. Although GSDMD was initially thought to be required for NET formation, studies have shown that GSDMD-deficient mouse neutrophils are as competent as wild-type mouse neutrophils in producing NETs. Furthermore, the process of generating NETs through both noncanonical and canonical inflammasome signaling pathways also does not require GSDMD (32). There is still much controversy and conflicting conclusions regarding the requirements of PAD4 activity for the formation of NETs (28, 33–35). Neutrophils from PAD4 knockout mice are unable to citrullinate histone H3 and fail to release NETs upon stimulation or infection by PMA, LPS, calcium ionophores, *Shigella flexneri*, or methicillin-resistant *Staphylococcus aureus* (MRSA). This study demonstrated the necessity of PAD4 in the formation process of NETs (36). However, there are also some different conclusions. For example, PAD4 is not necessary for the process of NETosis induced by *C. albicans* or group B Streptococcus infections (27). Additionally, PAD4 release and histone arginine citrullination can exist independently of NET formation (37). Overall, it should be recognized that there has been continuous debate regarding the process of neutrophil extracellular traps formation. This controversy arises from various stimuli including bacteria, viruses, fungi, or chemicals, as well as variations in neutrophil types and detection techniques. It could be valuable to investigate this subject under specific circumstances (Figure 1).

### 2.2 Vital/non-lytic NETosis

In addition to lytic NETosis, neutrophils can also release NETs through vesicular secretion, known as non-lytic NETosis. This process has been observed in neutrophils that come into contact with activated platelets, as well as in response to *S. aureus* and *C. albicans* infections. In non-lytic NETosis, neutrophils encapsulate NETs complexes in vesicles and transport them to the extracellular space for release, while the cells themselves remain viable and functional, maintaining the integrity of the cell membrane.

Various pathogens activate neutrophils to release NETs through distinct mechanisms. For example, *S. aureus* and *C. albicans* activate Toll-like receptor 2 (TLR2), whereas the LPS in Gram-negative bacteria (*Escherichia coli* and *Pseudomonas aeruginosa*)-induced Toll-like receptor 4 (TLR4) to initiate NETs release (38–40). What is different from the above process is that, platelet-induced NETs formation during infection is LFA1-reliable process and which also depends on the direct interaction of neutrophils and platelets (41). In non-lytic NETs release, similar to lytic NETosis, peptidylarginine deiminase 4 (PAD4) and neutrophil elastase (NE) also play roles in chromatin decondensation within the nucleus (3). Non-lytic NETs release represents a non-self-sacrificing anti-inflammatory mechanism of neutrophils, occurring more rapidly (within 5–60 min) compared to lytic NETsosis (3–4 h), resembling the initial response of neutrophils to infection (26). Many aspects of the pathological and physiological mechanisms of non-lytic NETs release remain unclear including vesicle formation for NETs transport, the process of NETs release, and the involved signaling pathways (Figure 1).

## 3 NETs in airway inflammatory diseases

### 3.1 Acute lung injury/acute respiratory distress syndrome

#### 3.1.1 Sepsis-associated ALI/ARDS

Sepsis is a syndrome characterized by a systemic inflammatory response caused by invading pathogenic microorganisms, such as bacteria. It has a high incidence and mortality rate among critically ill individuals. Acute respiratory distress syndrome (ARDS) or its early stage, acute lung injury (ALI), is a common complication of sepsis and one of the most critical prognostic factors for mortality in septic patients. ARDS/ALI is characterized by lung inflammation, increased microvascular permeability, lung edema, membrane formation, and interstitial fibrosis (42, 43). These pathological changes result in reduced lung compliance, increased intrapulmonary shunting, and ventilation-perfusion mismatch, ultimately leading to respiratory distress and severe hypoxemia. The migration and infiltration of activated neutrophils in lung tissue play a crucial role in the development of ARDS/ALI, causing damage to epithelial and endothelial cells, among other effects. Neutrophils are considered as critical inflammatory cells in the pathogenesis of ARDS. The emerging research evidence suggests that neutrophil extracellular traps released by neutrophils are involved in the pathological process of ALI/ARDS. This discovery provides important clues for further understanding the development mechanism and treatment strategies of ARDS, offering hope for improved care and treatment for patients.

NETs are an innate defense mechanism responsible for clearing pathogens during early infection and inflammation. However, excessive NETs can cause damage to host cells and tissues, making it crucial to regulate their balance (1). PAD4 plays a crucial role in the process of neutrophil releasing NETs by modifying histones and causing chromatin decondensation. Lefrançais et al. (44) constructed a mouse model of lung injury by intratracheal instillation of methicillin-resistant *S. aureus* (MRSA). The severity of lung injury is reduced in PAD homozygous knockout mice (PAD^−/−^), but bacterial load and levels of inflammatory cytokines increase. Ultimately, compared to wild-type mice, the survival rate of PAD^−/−^ mice does not show a significant improvement. However, the survival rate is obviously improved in PAD heterozygous knockout mice (PAD^+/−^), which have a phenotype intermediate between PAD^−/−^ and wild-type mice. Moreover, elevated levels of plasma NETs have been associated with the severity and mortality of sepsis-related ARDS, while lower levels of plasma DNase I have been associated with the development of sepsis-induced ARDS. Inhibitors of PAD2/PAD4, such as YW3-56, have demonstrated the ability to inhibit LPS-induced lung vascular leakage, decrease acute lung injury, and improve survival rates in mouse models of endotoxemia (45). Interestingly, it has been reported that in sepsis-related acute lung injury, the PAD2 inhibitor AFM32a can reduce the production of inflammatory factors, alleviate lung injury, and inhibit the generation of NETs. As a result, it improves survival rates (46). These findings suggest that both of PAD2 and PAD4 may play a significant role in the pathological process of sepsis-related lung injury. NE is one of the main components of NETs. Sivelestat is a selective NE inhibitor and the only specific therapy available for ARDS. A systematic review and meta-analysis revealed that sivelestat not only reduces the mortality rate and incidence of adverse events in ALI/ARDS patients, but also shortens mechanical ventilation time and ICU stay, increases non-ventilated days, and improves patient’s oxygenation index (47). However, the efficacy of sivelestat sodium in treating ARDS is still controversial in clinical research (48, 49). The use of DNase to degrade NETs related DNA is a therapeutic approach for treating lung injury mediated by NETs. Research with animal models has found that treatment with DNase I can alleviate inflammatory damage in the airways (15, 44).

Zhang et al. (50) reported that NETs induce ferroptosis in alveolar epithelial cells through the activation of METTL3-mediated m6A modification. Additionally, inhibiting NETs formation by knocking out peptidylarginine deiminase 4 (PAD4) can alleviate ferroptosis and sepsis-related lung injury in mice. There might be an association between NETs and pyroptosis. In a mouse model of ALI induced by LPS attack, the levels of NETs and the proportion of necrotic alveolar macrophages were significantly increased. The NETs-DNA degrading agent DNase I can alleviate alveolar macrophage necrosis, and the pyroptosis inhibitor Ac-YVAD-cmk can also alleviate the levels of NETs in BALF and neutrophil infiltration in the alveoli. Poly I:C can induce the production of NETs and the occurrence of lung injury. Inhibiting the receptor TLR3 of Poly I:C can suppress the aforementioned response. Additionally, Poly I:C induces the activation of p38 MAPK, and p38 inhibitor can inhibit the generation of NETs (51). Therefore, the TLR3-P38-MAPK pathway may become a potential target for regulating the production of NETs. IRF-1 is a transcription factor involved in regulating immune and inflammatory responses. It plays an important role in physiological and pathological states such as infection, tumor formation, and autoimmunity. In the LPS-induced ALI model, IRF-1 gene knockout mice showed significantly reduced lung injury and decreased release of NETs. In addition, IRF-1 plays a crucial role in regulating the classical ROS-dependent NETosis process (52). This provides another perspective for us to further understand the mechanism of NETs production and study their clinical application value. Our team has also previously discovered that NETs regulate LPS-induced acute lung injury through the cGAS-STING pathway, and that DNase or inhibitors of the cGAS-STING pathway can alleviate lung injury (53). In a mouse model of acute lung injury induced by mitochondria, neutrophils from older mice showed impaired chemotactic activity, but trended towards higher formation of extracellular traps (14). This may be related to age-related immune regulation.

Du et al. (54) have successfully developed a novel biomimetic DNase I delivery system called DCNV (genetically and bio-orthogonally engineered cell-derived nanovesicles). This innovative system demonstrates high efficiency in targeting the lungs during LPS-induced acute lung injury and effectively clears pulmonary NETs. This study suggests that we can enhance the therapeutic efficacy of drugs through materials engineering modifications. However, the safety of drugs or drug delivery systems needs further confirmation. Recently, research has reported the therapeutic effects of traditional Chinese medicine in this aspect. Lianhua Qingke (LHQK) can effectively ameliorates lung inflammatory response and lung injury partially by inhibiting cf-DNA, CitH3-DNA levels, the components of NETs (55). Xuebijing injection is a traditional Chinese medicine compound formulation that includes components such as safflor yellow A (SYA), hydroxysafflor yellow A (HSYA), and anhydrosafflor yellow B (AHSYB). Research has found that these components can inhibit the production of NETs *in vitro* or *in vivo*, and alleviate LPS-induced airway injury (56). However, the specific molecular mechanism is still not clear, and further research is needed to explore the interaction between Xuebijing injection and NETs, as well as its potential impact on the treatment of sepsis. In addition, Xuanfei Baidu decoction (XFBD) remarkably inhibit NETs markers MPO and H3Cit expression in LPS induced acute lung injury in mice, and meanwhile alleviate inflammatory lung injury (57). It is undeniable that further research is needed to clarify the efficacy, mechanisms of action, and specific active ingredients of traditional Chinese herbal medicines in the treatment of acute lung injury. In recent years, studies have shown that stem cell therapy has demonstrated certain potential value in research on various disease models, and some have even shown clinical applications (58). For example, mesenchymal stem cells from bone marrow can regulate lung inflammation, reduce oxidative damage, and decrease NETs release in mice with LPS-induced acute lung injury models, offering a promising therapeutic approach (59). An iron-chelating agent, deferasirox (DFS), can inhibit the generation of dsDNA or NETs in BALF induced by LPS, thereby alleviating lung injury, this may be partially attributed to the inhibition of ROS generation (60).

In summary, NETs represent the promising therapeutic targets in sepsis or endotoxin-related acute lung injury/ARDS and can be approached from various directions, such as increasing NETs clearance, reducing NETs production, or selectively inhibiting NETs and their components. While there is growing evidence suggesting the involvement and significance of NETs in airway inflammatory diseases, most research is still in the preclinical stage, involving cell and animal experiments. Further basic and clinical research is needed to fully understand the process of NETs formation, their biological characteristics, and regulatory role in the inflammatory micro-environment. This will help us identify potential therapeutic targets and develop targeted therapies that can effectively modulate their harmful effects while preserving their beneficial functions in host defense. It is crucial to transfer our comprehension regarding NETs from laboratory experiments to practical applications. Additionally, there is a need for further clinical research to assess the effectiveness and safety of therapies focusing on NETs in the treatment of ALI/ARDS and other respiratory disorders.

#### 3.1.2 Transfusion-related acute lung injury

Transfusion-related acute lung injury (TRALI) is a cause of death in critically ill patients following blood therapy, whether it involves whole blood or blood components. TRALI usually manifests within 6 h of transfusion and is clinically characterized by hypoxemia and respiratory distress. Pathologically, TRALI is characterized by pulmonary edema, congested capillary leukocytes, and diffuse infiltration of neutrophils in lung tissue (61, 62). Patients with TRALI have been found to have elevated levels of neutrophil extracellular traps in their lungs and plasma (63, 64). Caudrillier et al. (64) established a mouse model of neutrophil and platelet dependent TRALI, in which they observed a significant increase in NETs in both the pulmonary microcirculation and plasma. Pulmonary microthrombi in the lung vasculature are a key pathological feature of ARDS/ALI. Platelet activation can promote the release of NETs, and the histones expressed on NETs may, in turn, activate platelets, leading to further release of NETs and promoting pulmonary coagulation and thrombus formation, thus creating a vicious cycle. Furthermore, targeted inhibition of platelet activation using aspirin or glycoprotein IIb/IIIa inhibitors, or directly targeting components of NETs with histone-blocking antibodies and DNase I, has shown improved outcomes in terms of pulmonary edema, vascular permeability, and even mortality. Additionally, studies have investigated the use of small molecules like β-nitrostyrene derivatives (BNSDs), such as compound C7, which have demonstrated protective effects against LPS-induced acute lung injury. These compounds inhibit neutrophil aggregation, release of inflammatory mediators, platelet aggregation, myeloperoxidase activity, and release of NETs (65). The interactions between neutrophils and platelets in the pathophysiology of TRALI present a potentially modifiable target for drug interventions. Furthermore, studies have explored the mechanisms of NETs-mediated TRALI in mouse models. It has been discovered that increased expression of miR-144 promotes NETs-induced TRALI by down-regulating KLF2 (Krüppel-like factor 2) and activating the NF-kappaB/CXCR1 (C-X-C motif receptor 1) signaling pathway (66). We casually speculate these findings shed light on the molecular pathways involved in TRALI and provide potential targets for therapeutic interventions. Additionally, studies have reported the activation of the complement system in TRALI patients, which is associated with an increase in NETs. In an *in vitro* “two-hit model” of TRALI simulated by LPS and C5a, the formation of NETs was also observed (67). These findings suggest that the complement system may also be involved in the pathological process of TRALI.

In summary, although there is currently limited research on NETs-mediated TRALI, NETs have been implicated in the pathogenesis of TRALI, and understanding their role in this condition may help develop targeted therapies to prevent or treat TRALI in the future. However, it is important to note that further research is still needed to fully elucidate the mechanisms underlying NETs-mediated TRALI and evaluate the efficacy of potential interventions.

#### 3.1.3 Ventilator-associated lung injury

Mechanical ventilation is a commonly used supportive treatment in the ICU to assist patients with their breathing. Its primary goals are to ensure airway patency, enhance ventilation and oxygenation, and prevent hypoxia and hypercapnia. However, it is important to note that mechanical ventilation can also lead to lung injury, which is referred to as ventilator-associated lung injury (VALI). VALI can occur due to multiple factors, such as high tidal volumes, excessive airway pressure, repetitive alveolar collapse and recruitment, and the release of pro-inflammatory mediators. These factors can cause inflammation, oxidative stress, alveolar damage, and disruption of the lung’s physiological balance. Additionally, mechanical ventilation can contribute to lung overdistension and shear stress, further exacerbating lung injury (68–70). During mechanical ventilation, a significant release of inflammatory cytokines and chemokines occurs, leading to the recruitment of numerous neutrophils into lung tissues, where neutrophils are the primary effector cells in VALI (71–73). Studies have shown that in ventilator-induced lung injury models, the accumulation of neutrophils in the lungs can be observed early, even before physiological signs of lung injury become apparent. Additionally, in VILI models, the expression of chemokines like CXCL1 (KC) and CXCL2/3 (MIP-2) significantly increases, and their interaction with CXCR2 plays a crucial role in recruiting neutrophils and contributing to lung inflammation (74). These inflammatory factors such as IL-8, IL-1β, and TNF-α can also induce the release of neutrophil extracellular traps from neutrophils (75, 76). It has been proved the NETosis occurs in the pathogenesis of ventilator-associated lung injury, certainly, the specific mechanism is not clear.

In a mouse model of neutrophil and platelet-dependent VILI, increased of circulating NETs component MPO-DNA and the formation of NETs is required the simultaneous stimulation of integrins and G-protein-coupled receptors on neutrophils. Additionally, the heterodimerization of platelet-derived CCL5 and CXCL4 enhances their ability to activate and recruit inflammatory cells. Therefore, the study showed that blocking the heterodimerization of CCL5 and CXCL4, regulating NETs formation by DNase I, or inhibiting integrins/G-protein-coupled receptors could alleviated ALI (77). In a VILI model, the components of pulmonary NETs, including DNA, Cit-H3, and NE, were found to increase. Pre-treatment with DNase I to degrade NETs could alleviate previous lung injury. Furthermore, in a TLR4 knockout acute lung injury mouse model, NETs formation was reduced and lung injury was relieved. Therefore, the authors believe that TLR4 partially mediates the process of NETs promoting VILI (78). However, the authors believe that it is difficult to determine whether the above changes have a direct causal relationship with TLR4 or are just accompanying changes. These findings suggest that NETs may play an important role in VILI. Further research on the role of NETs in VILI and related pharmacological interventions may help us better understand the pathogenesis of VILI and provide new insights into its prevention and treatment. Although the mechanism by which NETs participate in ventilator-associated lung injury is unclear, it is undeniable that NETs and their components, or the pathways they generate, may become targets for the treatment of lung injury.

### 3.2 NETs in asthma

Bronchial asthma is a chronic inflammatory disease characterized by heterogeneous inflammation involving multiple cell types, such as eosinophils, mast cells, T lymphocytes, neutrophils, and airway epithelial cells. Asthma patients experience variable and reversible airflow obstruction, often presenting with recurrent wheezing, shortness of breath, chest tightness, and/or cough (79–81). The traditional view recognizes eosinophilic inflammation as a characteristic feature of the pathogenesis of asthma. However, current evidence confirms the importance of neutrophilic inflammation in asthma, and in certain cases, eosinophils and neutrophils can coexist, with neutrophils even playing a predominant role. Asthma phenotypes are commonly classified based on the cellular profile obtained from induced sputum. Additionally, methods such as nasal lavage or nasal irrigation are also used for assessing asthma subtypes.

Neutrophil-mediated asthma airway inflammation differs from Th2 cell-mediated eosinophilic airway inflammation, and is referred to as Th2-low asthma (82, 83). The active involvement of neutrophils can lead to airway inflammation and pathological changes. Compared to Th2 cell-mediated asthma, Th2-low asthma patients have lower levels of IgE and fewer eosinophil, and may not be responsive to traditional asthma treatments such as corticosteroids. It should be noted that Th2-low asthma and Th2-high asthma are not strictly mutually exclusive, and some patients may have both types of airway inflammation (84). Currently, there is much debate regarding the existence of neutrophilic asthma. For example, it has been argued that the elevated levels of neutrophils in the airways of asthmatic patients could be a result of corticosteroid treatment, as glucocorticoids can enhance neutrophil survival and neutrophils express glucocorticoid receptor β (GRβ) (85). However, increased neutrophil levels have also been observed in asthma patients who have not received steroid treatment (86). Therefore, in addition to the traditional eosinophilic inflammation, neutrophilic inflammation also plays an important role in asthma, and there are instances where neutrophils may even surpass eosinophils in quantity.

The high expression of NETs has been found in bronchial biopsy specimens, sputum, and plasma samples from asthma patients (87–89). Particularly, there is a correlation between high levels of extracellular DNA (eDNA) in sputum and severe asthma (87). In neutrophilic asthma patients, there is an elevation in extracellular DNA levels in sputum, and it is negatively correlated with FEV1% (90). The increase in eDNA in sputum of asthma patients is associated with disease severity, as well as the increased frequency of oral corticosteroid use. And there is a correlation between the increased presence of eDNA and NETs components NE-DNA and H3Cit-DNA (91). The plasma levels of CitH3 are elevated in asthma patients, and the concentration is negatively correlated with the percentage decrease in FEV1/FVC in asthma patients (92). Furthermore, in models of asthma induced by viral infection, LPS, HDM, and air pollutants, the involvement of NETs has been observed (93–95). One of the components of NETs Double-stranded DNA (dsDNA) promote rhinovirus-induced type-2 allergic immune responses and asthma exacerbation (93). These studies suggest that NETs are involved in the pathogenesis of asthma and may even be associated with the severity or acute exacerbation of asthma.

Unlike with the Th2-dominated eosinophilic asthma, neutrophilic asthma is more obviously associated with the presence of Th17 cells and their related cytokines such as IL-17 (91, 96–98). In children with exacerbation of asthma, there is a significant increase in IL-17A levels in their sera. *In vitro* studies using stimulated neutrophils from children with asthma exacerbation have shown that it can induce the formation of IL-17A-enriched NETs. These IL-17A-enriched NETs, in turn, promote fibrotic changes *in vitro* (99). This suggests a potential role of IL-17A/NETs in the pathogenesis of asthma exacerbation and associated fibrosis. In patients of severe asthma, particularly in patients with a neutrophil proportion of ≥5%, BALF DNA is significantly increased, and high expression of H3Cit-is also observed in severe asthma with a higher proportion of neutrophils, and NETosis is associated with IL-17 levels. Meanwhile, in animal models, exposure of allergen-sensitized mice to LPS can lead to NETosis in lung tissue and mediastinal lymph nodes. In PAD4 knockout mice, NETosis is reduced and Th17 inflammatory responses are decreased (97). Which indicate that NETosis may play an immunomodulatory role in Th17/IL-17-mediated severe asthma. Recent clinical studies have shown that blocking the IL-33 receptor chain ST2 can reduce the frequency of uncontrolled acute exacerbations in type 2 low asthma patients who have fewer eosinophils in their peripheral blood (100). In addition, anti-IL-33 has been shown to inhibit neutrophilic inflammation in animal models of asthma. These findings suggest that IL-33 may be involved in non-type 2 inflammation (101). As previously mentioned, rhinovirus can cause acute exacerbation of asthma, and a team of researchers explored its mechanism and found that blocking IL-33 can reduce the aggregation of neutrophils and the formation of NETs, ultimately alleviating acute exacerbations in asthma (102). Recently, Tsai et al. (103) reported the association between NETs and the asthmatics who was nonresponse to inhaled corticosteroids, or termed uncontrolled asthma. Furthermore, they found the DNase I could significantly inhibited airway hyperreactivity and inflammation, even in the murine model of steroid resistant neutrophilic airway inflammation. CCL4L2 was observed associated with the insensitive effect of steroids treatment in asthmatics and mouse model. Interestingly, NE-deficient mice showed impaired NETosis in asthma models induced by OVA and LPS, accompanied by decreased levels of dsDNA and expression of inflammatory factors such as IL-1β, IL-6, and IL-17. Collectively, these results indicate the involvement of NETs in neutrophil-mediated airway inflammation and may also suggest the potential therapeutic target of CCL4L2 for asthmatics refractory to steroids. These results suggest that NETs may be involved in steroid-resistant asthma. In the neutrophilic asthma model induced by OVA/CFA/LPS, researchers have found that NETs are involved in the pathological process of asthma (104). In the neutrophilic asthma model, treatment with DNase I or CI-amidine can reduce the expression of MPO and CitH3, decrease airway hyperresponsiveness, alleviate the accumulation of inflammatory cells and mucus obstruction, and mitigate inflammation-induced damage. In Th17-mediated severe asthma models induced by OVA/HDM/LPS, characterized by neutrophilic aggregation, the expression of Th2 cells, Th17 cells, and their associated inflammatory factors increases. The expression of NETs-associated dsDNA, MPO-DNA, CitH3, and PAD4 also increases. Treatment with Simvastatin can suppress the inflammatory response and reduce NETs generation. Mechanistic studies suggest that this therapeutic effect of Simvastatin may be achieved through the inhibition of PAD4 (105).

During the inflammatory process, damaged or infected tissues release chemokines, such as interleukin-8 (IL-8), which are CXC chemokines. These chemokines can bind to specific receptors on the surface of neutrophils, such as CXCR1 and CXCR2, activating the movement and chemotactic response of neutrophils (106). CXCR2 antagonist AZD5069 is being studied for reducing the number of neutrophils in the sputum of severe asthma patients, however there is no reduction in the frequency of severe exacerbations (107–109). However, the clinical effectiveness of CXCR2 antagonists in the treatment of asthma is currently uncertain. For instance, in patients receiving SCH527123 treatment, there may be a reduction in mild exacerbations (*p* = 0.05) and improvement in ACQ scores (*p* = 0.053), and there is no statistically significant difference in FEV1 changes (109). This could be attributed to the complexity of asthma, which involves interactions among multiple inflammatory cells and mediators. Targeting solely neutrophils may not fully address the intricate pathological mechanisms of asthma. Although the relationship with NETosis remains unclear, these findings offer a potential indication of whether CXCR2 antagonists can be utilized to inhibit neutrophils and modulate NETosis for therapeutic benefits.

Recombinant human deoxyribonuclease I (rhDNase) intervention in the OVA-induced eosinophilic asthma model also reduces reactive oxygen species, increases the superoxide dismutase/catalase ratio, enhances glutathione peroxidase activity, and increases thiol content, thus significantly improving lung tissue oxidative stress (110). In a toluene diisocyanate induced animal model of asthma, Th2-related inflammatory factors and NETs levels increase. These can be suppressed by DNase I, but not by dexamethasone. In addition, inhibiting the expression of NETs can promote repair of damaged epithelium (111). Therefore, NETs may play a role in both eosinophil-and neutrophil-mediated asthma as they have been observed in models and clinical samples of both types of asthma. However, there is a lack of clear research evidence regarding the specific mechanisms of NETs in Th2-dominant allergic airway inflammation.

### 3.3 NETs in chronic obstructive pulmonary disease

Components related to NETs, such as cf-DNA, MPO, NE, LL-37 and a-defensins have been found to be elevated in the airways of chronic obstructive pulmonary disease (COPD) patients, suggesting their possible involvement in airway inflammation and tissue damage. COPD is a common and treatable inflammatory disease characterized by progressive airflow limitation and tissue destruction (112, 113). Stimuli such as cigarette smoke, environmental pollutants, bacteria, viruses, and oxidative stress induce the secretion of chemokines and inflammatory mediators by lung epithelial cells and macrophages, which in turn attract neutrophils and monocytes from the circulation to infiltrate the lung tissue, causing sustained inflammation (114–117).

It is suggesting that NETs formation is not limited to COPD exacerbations but also exists in stable COPD and is associated with the severity of airflow limitation (118). In a study based on COPD patients, it was found that the content of histone-elastase complex concentration in sputum is significantly correlated with severity indicators of COPD such as GOLD score, COPD assessment test (CAT) score, exacerbation frequency, and percent predicted FEV1. Other NET markers, such as cf-DNA, were not associated with exacerbation frequency or GOLD scores, while elastase, MPO, and EN-RAGE were associated with exacerbation frequency, percent predicted FEV1, and GOLD scores (119). The key component NE of NETs is associated with bacterial infection-induced acute exacerbations in COPD, and NE may become an accurate predictor of a bacteria-associated exacerbation (120). As described earlier, IL-8 can promote the process of NETosis through its receptors, CXCR1 and CXCR2, on neutrophils. Peripheral blood neutrophils from COPD patients do not spontaneously form NETs *in vitro*, but can form NETs upon stimulation with autologous sputum supernatant, the NETs formation could be reduced by CXCR2 antagonist AZD5069 (121). These results indicate the importance of the inflammatory environment in the process of NETosis, and suggest that CXCR2 may regulate the production of NETs. Besides, there is generation of NETs in the subacute airway inflammation mouse model induced by CS (cigarette smoke), and *in vitro* cigarette smoke extract (CSE) can stimulate the production of NETs in airway epithelial cells in an NADPH oxidase-dependent manner (122). When used in combination with tiotropium, neutrophil elastase inhibitor AZD9668 did not show any clinical benefits in COPD patients and had no impact on inflammatory biomarkers (123).

Under physiological conditions, NETs are degraded by endogenous nucleases and cleared by alveolar macrophages (124, 125). It has been suggested a significant reduction in the number of macrophages in sputum samples from COPD patients compared to normal controls, and the phagocytic function of surviving macrophages is also defective (87, 126). Therefore, we speculate that, the decreased ability of COPD patients to naturally clear NETs results in their accumulation in lung tissues, promoting inflammation and exacerbating lung injury. NETs are associated with the worsening of COPD, and selective inhibition of NETs formation or targeting components may become potential therapeutic approaches for alleviating COPD. The specific mechanisms of how NETs are involved in the pathophysiology of COPD are still unclear and further basic experiments and clinical trials are needed for in-depth research.

### 3.4 NETs in COVID-19 related ARDS

In COVID-19, ARDS occurs as a result of an excessive immune response to the viral infection. The release of pro-inflammatory cytokines and the recruitment of immune cells, including neutrophils, contribute to the formation of NETs. The presence of a neutrophil activation signature is a prominent characteristic observed in the transcriptomes of circulating leukocytes in severe cases of COVID-19. NETs released during COVID-19 can cause direct damage to lung tissue and induce further inflammation. They can also contribute to the formation of microthrombi, impairing blood flow and oxygenation in the lungs. Additionally, the excessive presence of NETs can activate immune cells and amplify the immune response, leading to a cytokine storm and worsening lung injury.

The findings of the prospective cohort study indicate a significant increase in CitH3 and MPO in COVID-19 patients who require endotracheal intubation. The ratio of Pao_2_/FiO_2_ inversely correlates with the levels of NETs. Additionally, there is a direct correlation between plasma NETs and the Sequential Organ Failure Assessment (SOFA) score, which is a clinical marker used to assess the severity of illness. The increased levels of CitH3 and MPO, which are associated with NETs, further support the involvement of NETs in the pathogenesis of COVID-19 related ARDS (127). The levels of cf-DNA, MPO-DNA, and CitH3 are elevated in the serum of COVID-19 patients. Besides, hospitalized patients who require mechanical ventilation exhibit higher levels of cf-DNA and MPO-DNA (128). These findings suggest that the presence and abundance of NETs in the plasma of COVID-19 patients may serve as indicators of disease severity and organ dysfunction. There have been studies investigating the underlying mechanisms of NETs in mediating COVID-19 related ARDS. Indeed, SARS-CoV-2 has the ability to directly stimulate human neutrophils to release NETs. This stimulation occurs through the interaction of the viral spike glycoprotein (S) with angiotensin-converting enzyme 2 (ACE2) and transmembrane serine protease 2 (TMPRSS2) (29). ACE2 is known to facilitate viral entry into host cells and is expressed in various cell types, including lung pneumocytes, epithelial cells, and endothelial cells (129). The activation of ACE2 by SARS-CoV-2 plays a critical role in triggering the release of NETs by neutrophils. NETs-based immunothrombosis is another critical mechanism for disease severity or organ damage (130). Autopsies of the lungs of COVID-19 patients have shown diffuse neutrophil infiltration accompanied by extensive NET deposition, often associated with platelet aggregation and microvascular thrombosis (131). This finding suggests that the formation of microthrombi may be associated with the severity of COVID-19 in patients. The formation of microthrombi can lead to impaired pulmonary circulation, exacerbate lung injury, and further worsen the condition of COVID-19. Studies have shown that NETs can promote thrombus formation through various mechanisms, including platelet adhesion and activation, binding to cells and fibrinogen, and interaction with von Willebrand factor (vWF). NETs-driven thrombosis is primarily platelet-dependent, and have been suggested as a crucial contributor to neutrophil-related thrombo-inflammation (132). NETs also contribute to the generation of local clotting enzymes, increasing the likelihood of clot formation. They can activate the extrinsic pathway through the production of tissue factor (TF) and initiate thrombus formation by activating the contact pathway via the activation of coagulation factor XII (FXII) (133). Certainly, the formation of pulmonary microthrombi mediated by NETs is a complex cascade reaction involving the interaction of various factors, including cytokines, chemokines, platelets, hemostatic and coagulation systems (134). Given the prominent role of immune thrombosis in the pathogenesis of COVID-19-associated ALI/ARDS, pharmacological inhibition or degradation of NETs may be a promising approach to alleviate disease severity and improve survival rates. In the treatment of COVID-19 ARDS, specific strategies targeting NETs have not been widely adopted. Although NETs play a role in inflammation and thrombus formation, there are currently no specific drugs or treatment methods available to specifically target NETs.

### 3.5 NETs in pulmonary cystic fibrosis

Pulmonary cystic fibrosis (PCF) is a commonly inherited disease caused by mutations in the cystic fibrosis transmembrane conductance regulator (CFTR) gene. PCF is a recessive genetic disorder. CFTR gene mutations result in the dysfunction of chloride ion transport across the respiratory epithelium, which plays a crucial role in the formation of thin, freely flowing mucus. Therefore, patients with PCF will produce a large amount of thick mucus in their lungs, blocking the airways and bronchioles at various levels, leading to bacterial colonization. Pathophysiological studies have confirmed that cystic fibrosis is primarily driven by neutrophils (135–138). Mutations in the CFTR gene in individuals with cystic fibrosis make them more susceptible to respiratory infections, such as *P. aeruginosa*. The recurring infection process leads to a significant infiltration of neutrophils in the lungs, triggering the occurrence of chronic inflammation. Meanwhile, once infected, neutrophils are attracted to the airways, resulting in the formation of NETs that worsen the disease. The sputum of PCF patients contains NETs-related proteins such as NE and MPO, as well as neutrophil-associated proteins. Additionally, it contains a high concentration of extracellular DNA, which originates from necrotic neutrophils (139–141). NETs contribute to the thickening of mucus in the airways of CF patients, and enzymes like NE and MPO cause damage to the respiratory epithelium and connective tissue (140, 142–144). In the key components of NETs, MPO is believed to be associated with the severity of diseases and decline in lung function, while NE is considered a biomarker for the severity of CF disease and also promotes disease progression (145, 146). Calcium-binding protein S100A8/A9 has recently received significant attention as a NET protein, which has been found in the sputum, serum, and bronchoalveolar lavage of CF patients (147–149). Its expression levels can serve as biomarkers for CF patients and are associated with lung function decline (150). Our previous research has confirmed that neutrophils in the inflammatory environment of the airways have an extended lifespan, which sustains the persistent presence of inflammation (151). Similarly, in patients with cystic fibrosis, the longer lifespan of neutrophils leads to an increased production of NETs (152). This may, at least partially, explain how NETs contribute to the progression of CF disease. These studies all indicate that NET and component proteins play a crucial role in lung CF, and targeted treatment against NET may have potential clinical benefits for pulmonary CF patients. Nebulized inhalation of rh-DNase I (pulmozyme) is a clinical method for treating patients with cystic fibrosis. rh-DNase I can break down excessive DNA in the respiratory secretions of CF patients, thereby reducing the amounts of respiratory secretions and the risk of respiratory tract infection, and improving lung function in patients. In addition, studies have shown that the use of rh-DNase I not only rapidly improves FEV1, but also correlates with improvement in the rate of FEV1 decline within 2 years of long-term use (136, 153, 154). Not all drugs are effective, for example, CXCR2 antagonists named SB-656933 did not provide clinical benefits for CF patients (155). CF patients received neutrophil elastase inhibitor AZD9668 suggested no impact on sputum neutrophil count, neutrophil elastase activity and lung function. However, there is a decreasing trend in inflammatory biomarkers in sputum (156). Similar to other respiratory diseases, further research is still needed to understand the specific molecular mechanisms through which NETs mediate diseases and explore the feasibility of targeting them for treatment due to the complex composition of NETs.

## 4 Crosslinking between macrophage and NETs

Macrophages play a crucial role in the pathogenesis of inflammation by participating in inflammation, immune regulation, and post-injury repair processes. Macrophages are highly plastic cells that can polarize into several distinct subtypes and exert different functions under different stimuli (157, 158). It has been reported that NETs can promote the polarization of macrophages towards the M1 phenotype, thereby facilitating acute-phase inflammation in ARDS (159). Indeed, we also know that excessive polarization of M2 macrophages can lead to the progression of pulmonary tissue fibrosis in the late stage of ARDS. Therefore, it is worthwhile to explore the targeting of NETs levels and macrophage polarization status in conducting ARDS/ALI-related research. A large amount of NETs has direct cytotoxicity on epithelial cells and is engulfed by alveolar macrophages, leading to AIM2 inflammasome activation and caspase-1 dependent cell pyroptosis in alveolar macrophages, followed by the release of more inflammatory factors, inducing more neutrophil infiltration (160). In a sepsis-induced lung injury model, researchers found that increased NETs can promote the assembly of NLRP3 inflammasome, activate caspase-1, and ultimately lead to alveolar macrophage pyroptosis. However, this phenomenon was not observed after degradation of the NETs. Besides, NETs induce an increase in ROS, which promotes the activation of NLRP3 NLRP3 deubiquitination, leading to subsequent pyroptosis in alveolar macrophages. Inhibition of ROS can inhibit the NLRP3 pathway and further alleviate inflammation-induced lung injury (161).

In ARDS patients, the ability of macrophages to phagocytose NETs and apoptotic neutrophils is diminished. These NETs further activate inflammatory responses and induce fibrosis and lung atelectasis, thereby exacerbating lung injury (162). These effects may be due to abnormal macrophage function or inhibition by inflammatory factors. In addition, metformin can activate AMPK activity in bronchoalveolar lavage (BAL) macrophages of ARDS patients, enhancing their ability to uptake apoptotic neutrophils and NETs. This enhanced uptake capacity of macrophages may help alleviate lung injury caused by ARDS (162). In a study on endotoxemia-induced acute lung injury, it was found that metformin alleviates acute lung injury caused by endotoxemia by restoring AMPK-dependent mTOR inhibition (163). Therefore, metformin may have the potential as a therapeutic agent for treating ARDS. However, further research is needed to validate its efficacy and ensure its safety. *In vitro* experiments have found that human granulocytes can produce NETs upon cytokines stimulation, and co-culture with different immune cells revealed that macrophages can reduce the formation of NETs while NK cells or Treg cells have no effect (164). This further suggests that macrophages may be involved in the dynamic changes of NETs.

Currently, there is still much to be explored regarding the association between macrophages and NETs. Moreover, further investigation and research are needed to fully understand the role of NETs and potential therapeutic targets in other diseases.

## 5 Conclusion

**Figure 2:**
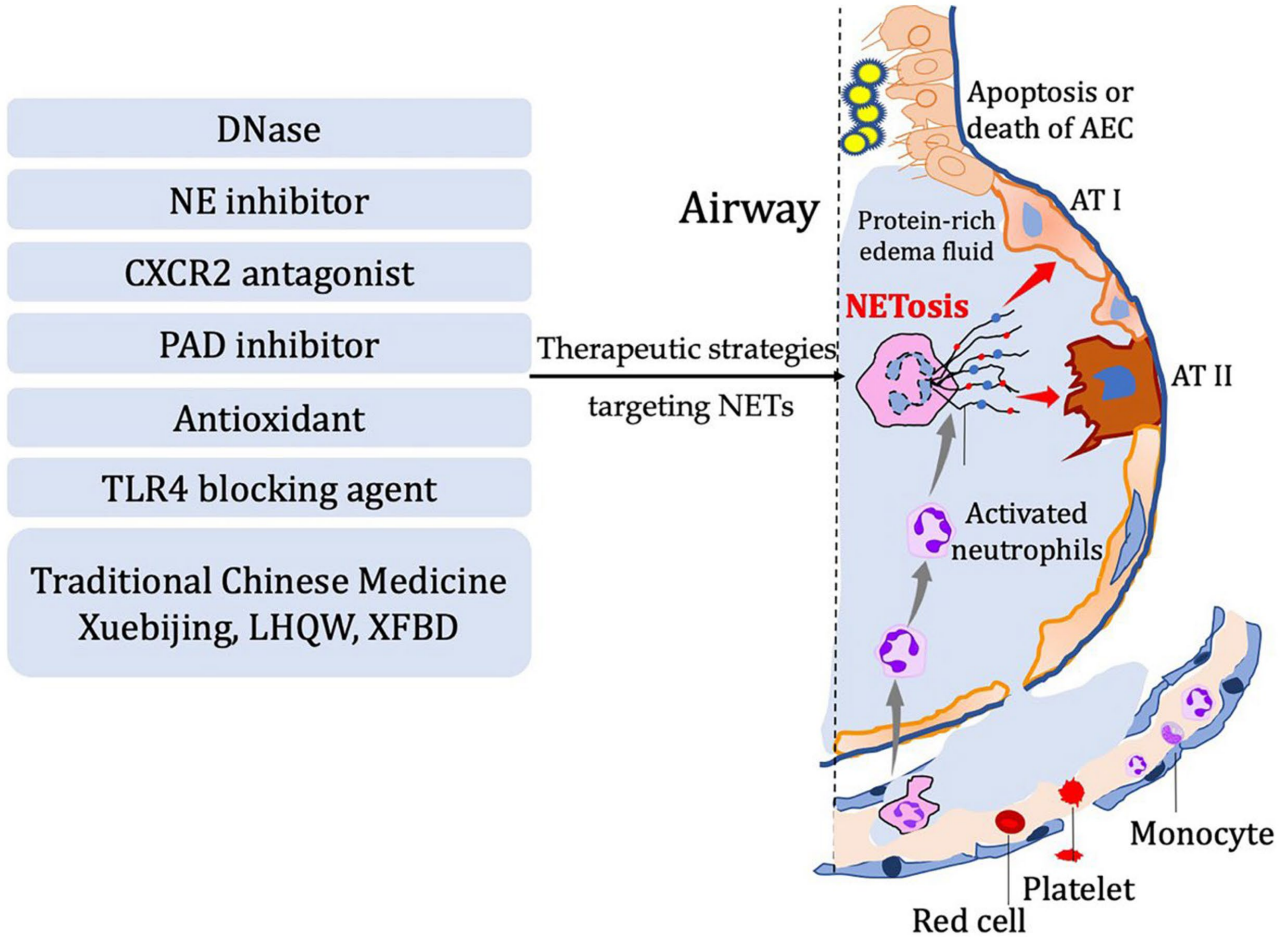
In diseases characterized by airway inflammation and injury, the activation of NETosis and the production of corresponding components mediate the inflammatory pathological processes in the lungs. Several potential therapeutic strategies have been developed based on the process of NETosis. These include DNase, NE inhibitor, CXCR2 antagonist, PAD inhibitor, antioxidants, TLR4 blocking agents, and even traditional Chinese herbal medicine.

Inflammation response is the body’s primary reaction to injury in order to restore internal homeostasis. However, excessive inflammation can actually lead to tissue damage. The role of NETs in the pathological processes of diseases effectively illustrates this viewpoint (Figure 2). Indeed, neutrophil extracellular traps (NETs) play a critical role in the inflammatory response by capturing and eliminating pathogens such as bacteria and viruses. These structures consist of proteins and other cellular components released by neutrophils, forming a mesh-like substance that can trap and kill pathogens. Additionally, NETs can release chemokines, cytokines, and other inflammatory mediators to attract and activate immune cells, thereby amplifying the inflammatory response. However, it is important to note that NETs can also contribute to tissue damage and exacerbate inflammation. They have the potential to induce cell death and disrupt cell membranes, leading to increased inflammation and tissue injury. Therefore, maintaining a balance and regulating the release and function of NETs is crucial for immune and inflammatory homeostasis. Neutrophil extracellular traps, have been found to play a crucial role in respiratory system inflammatory diseases, including acute lung injury/acute respiratory distress syndrome, chronic obstructive pulmonary disease, asthma, and cystic fibrosis. By examining recent research advancements, we can gain a better understanding of the development and mechanisms behind these diseases. This understanding opens up possibilities for the identification of novel therapeutic targets, offering potential avenues for managing these respiratory conditions (Table 1).

**Table 1:** NETs as the target for treatment of airway inflammatory disease.

Inhibitor	Target	Effect	Disease/animal model
DNase	NETs-DNA	Decrease protein leakage/pulmonary edema, reduce accumulation of inflammatory cells, alleviate inflammatory damage	ALI model
DNase	NETs-DNA	Alleviate lung injury	VILI model
DNase	NETs-DNA	Reduce NETs, inhibit airway hyperreactivity and inflammation, even in the steroid resistant neutrophilic airway inflammation	Asthma model
rhDNase	NETs-DNA	Reduce ROS, increase superoxide dismutase/catalase ratio, enhance glutathione peroxidase activity, increase thiol content, improve lung tissue oxidative stress, promote repair of damaged epithelium	Asthma model
rhDNase	NETs-DNA	Break down excessive DNA in the respiratory secretions, reduce the amounts of respiratory secretions and the risk of respiratory tract infection, and improve lung function	CF patient
Sivelestat	NE	Reduce the mortality rate and incidence of adverse events, shorten mechanical ventilation time and ICU stay, increase non-ventilated days, improve oxygenation index	ALI/ARDS patient
AZD9668	NE	No impact on sputum neutrophil count, neutrophil elastase activity and lung function, a decreasing trend in inflammatory biomarkers in sputum	CF patient
AZD9668	NE	No clinical benefits and no impact on inflammatory biomarkers	COPD patient
YW3-56	PAD2/PAD4	Inhibit lung vascular leakage, decrease acute lung injury, improve survival rates	LPS induced ALI
AFM32a	PAD2	Reduce the production of inflammatory factors, alleviate lung injury, inhibit the generation of NETs, improves survival rates	Sepsis-related ALI
Simvastatin	PAD4	Suppress the Th17-mediated neutrophilic inflammation, reduce NETs generation	Severe asthma model
Cl-amidine	Nonselective PAD inhibitor	Decrease citrullinated histone-DNA conjugates in BALF and NETs in plasma, fail to decrease NE-DNA complexes in lung, no effect on lung injury or inflammatory cell recruitment	Severe bacterial pneumonia/acute lung injury model
Cl-amidine	Nonselective PAD inhibitor	Decrease expression of MPO and CitH3, reduce airway hyperresponsiveness, alleviate cellular accumulation and mucus obstruction in airway inflammation, and mitigate inflammatory damage	Neutrophilic asthma model
Ac-YVAD-cmk	Pyroptosis	Alleviate levels of NETs in BALF and neutrophil infiltration in alveoli	LPS induced ALI
Deferasirox	Iron-chelating	Inhibit the generation of dsDNA and ROS, alleviate lung injury	LPS induced ALI
Compound C7	Neutrophil function of superoxide generation and elastase release	Inhibit neutrophil/platelet aggregation, inflammatory mediators, reduce release of MPO and NETs	LPS induced ALI
AZD5069	CXCR2	Reduce sputum neutrophil counts, no effect associated with improvement in clinical outcomes	Asthma patient
SCH527123	CXCR2	Reduce mild exacerbations and improve ACQ scores, no statistically significant difference in FEV1 changes	Asthma patient
AZD5069	CXCR2	Reduce the NETs formation	Peripheral blood neutrophils from COPD patient
SB-656933	CXCR2	No effect	CF patient
Metformin	AMPK	Enhance BALF macrophage ability to uptake apoptotic neutrophils and NETs	ARDS patient
LHQK	Uncertain	Reduce cf-DNA, CitH3-DNA levels, ameliorate inflammatory response	LPS induced ALI
Xuebijing	Uncertain	Inhibit the production of NETs, alleviate airway injury	LPS induced ALI
XFBD	Uncertain	Inhibit MPO and H3Cit expression, alleviate inflammatory lung injury	LPS induced ALI
MSC	Uncertain	Reduce oxidative damage, decrease NETs release	LPS induced ALI

ALI, acute lung injury; ARDS, acute respiratory distress syndrome; ACQ, asthma control questionnaire; CF, cystic fibrosis; COPD, chronic obstructive pulmonary disease; LPS, lipopolysaccharide; LHQK, Lianhua Qingke; MSC, mesenchymal stem cells; NETs, neutrophil extracellular traps; NE, neutrophil elastase; VILI, ventilator-induced lung injury; XFBD, Xuanfei Baidu decoction.

## Author contributions

NX: Validation, Writing – original draft, Writing – review & editing. JZ: Validation, Writing – original draft, Writing – review & editing. ZK: Writing – original draft. WC: Validation, Writing – review & editing. B-pT: Conceptualization, Investigation, Supervision, Validation, Writing – original draft, Writing – review & editing.
